# Fearful Aggravation Caused by Laurocerasus^200^

**Published:** 1892-01

**Authors:** George Wigg

**Affiliations:** East Portland, Oregon


### FEARFUL AGGRAVATION CAUSED BY LAUROCERASUS $^{200}$.

GEORGE WIGG, M. D., EAST PORTLAND, OREGON.

Miss May S., aged seven years, had been troubled for some time with an almost incessant cough. Many remedies had been given without any benefit resulting. When I saw the child it was my opinion that Laurocerasus was the remedy, so I put ten drops of the 200th potency into five tablespoonfuls of water, with instructions to take one teaspoonful every two hours when awake. This was at four P. M. After the fourth dose she fell asleep. At three A. M. she awoke in a very excitable condition. Her mother asked if "she had been dreaming," but her tongue was so stiff that she could not answer. All of a sudden she began to tremble all over as if in a chill. After about ten minutes she commenced to twitch and jerk. Her mother, thinking she was going into convulsions, sent for me. When I saw the child I certainly thought her in spasms. I asked if she was in pain, but owing to the tongue being thick or heavy, she could not articulate. The mind was clear. Thinking that the remedy might be the cause of the rumpus, I gave her Camphor, and soon after a cup of coffee. She came out all right in a few hours. Four months after this I was at the house, when her mother said to me: "Doctor, is it not strange that May has not coughed since she was threatened with convulsions?" It is now ten months since the above took place, and that cough has not returned. Yet there was nothing in that potency? Oh! no! Neither is there anything in that wire overhead on yon street. Yet that wire broke, and the result was, a span of dead horses, a dead driver, and a singed tom-cat. Yet there was nothing in it? Oh! no!

